# *Tobamovirus* infection aggravates gray mold disease caused by *Botrytis cinerea* by manipulating the salicylic acid pathway in tomato

**DOI:** 10.3389/fpls.2023.1196456

**Published:** 2023-06-12

**Authors:** Rupali Gupta, Meirav Leibman-Markus, Daniela Weiss, Ziv Spiegelman, Maya Bar

**Affiliations:** Department of Plant Pathology and Weed Research, Agricultural Research Organization, Volcani Institute, Rishon LeZion, Israel

**Keywords:** *Botrytis cinerea*, tobamovirus, ToMV, ToBRFV, salicylic acid, immunity

## Abstract

*Botrytis cinerea* is the causative agent of gray mold disease, and infects more than 1400 plant species, including important crop plants. In tomato, *B. cinerea* causes severe damage in greenhouses and post-harvest storage and transport. Plant viruses of the *Tobamovirus* genus cause significant damage to various crop species. In recent years, the tobamovirus tomato brown rugose fruit virus (ToBRFV) has significantly affected the global tomato industry. Most studies of plant-microbe interactions focus on the interaction between the plant host and a single pathogen, however, in agricultural or natural environments, plants are routinely exposed to multiple pathogens. Here, we examined how preceding tobamovirus infection affects the response of tomato to subsequent infection by *B. cinerea*. We found that infection with the tobamoviruses tomato mosaic virus (ToMV) or ToBRFV resulted in increased susceptibility to *B. cinerea*. Analysis of the immune response of tobamovirus-infected plants revealed hyper-accumulation of endogenous salicylic acid (SA), upregulation of SA-responsive transcripts, and activation of SA-mediated immunity. Deficiency in SA biosynthesis decreased tobamovirus-mediated susceptibility to *B. cinerea*, while exogenous application of SA enhanced *B. cinerea* symptoms. These results suggest that tobamovirus-mediated accumulation of SA increases the plants’ susceptibility to *B. cinerea*, and provide evidence for a new risk caused by tobamovirus infection in agriculture.

## Introduction

Throughout their life cycle, plants are continuously challenged by various types of pathogens. To cope with these challenges, plants evolved a two-tiered immune system that enables them to mitigate the effects of invading pathogens (Jones and Dangl, 2006; Delplace et al., 2020). Following pathogen infection, plants can develop induced resistance to cope with future attacks. Induced resistance is generally divided into systemic acquired resistance (SAR) and induced systemic resistance (ISR) (Walters and Heil, 2007). SAR, commonly triggered by local infection, can provide long-term resistance to subsequent infection (Klessig et al., 2018). SAR generally relies on salicylic acid (SA), which has been shown to increase in plant tissues during pathogenesis (Yalpani et al., 1991).

Countless studies in the past few decades have focused on elucidating the interactions between various plant hosts and the pathogens that threaten them, however, most of these studies investigate the interaction between the plant host and a single pathogen, in controlled conditions. In agricultural or natural environments, plants are routinely exposed to more than one pathogen at a given time. Such infections can be challenging to mimic in research settings. Some studies have attempted to elucidate the nature of particular diseases caused by a combination of pathogens (Lamichhane and Venturi, 2015; Fang et al., 2021; Tan et al., 2021). In some cases, one pathogen can prime immunity against another (Orton and Brown, 2016), while in other cases, a prior or simultaneous pathogen infection will increase plant susceptibility towards additional pathogens (Spoel et al., 2007; Cooper et al., 2008; Zhang et al., 2022b).

Plant viruses of the genus *Tobamovirus* (family *Virgaviridae*) are some of the most economically important plant pathogens. Tobamoviruses are highly infectious, and cause significant damage to various crop species. They are transmitted by mechanical contact: through e.g., workers’ hands, agricultural tools and soil (Broadbent, 1976), and the diseases symptoms caused by these viruses include stunting, leaf blistering, malformations and yellow mosaic patterns, fruit spotting and marbling and suppression of root branching (Luria et al., 2017; Vaisman et al., 2022). The tomato mosaic virus (ToMV) was isolated in the early 20^th^ century as a virus that severely impacts tomato crops (Broadbent, 1976). Since then, effective ToMV-resistance genes have been discovered in tomato, and currently most modern cultivars are immune to this virus (Hall, 1980). In recent years, the tobamovirus tomato brown rugose fruit virus (ToBRFV) (Zhang et al., 2022a) has caused significant damage to tomato crops, leading to reductions in fruit quality and yield in various parts of the world. This is largely due to the ability of the ToBRFV movement protein to overcome the durable tomato resistance gene, *Tm-2^2^* (Luria et al., 2017; Hak and Spiegelman, 2021). Since there is currently no available commercial genetic resistance to ToBRFV, the only means to control ToBRFV is by effective detection, containment and disinfection (Oladokun et al., 2019; Panno et al., 2019; Davino et al., 2020; Alon et al., 2021; Samarah et al., 2021; Dombrovsky et al., 2022).

In many cases, viral infection has been reported to exacerbate the symptoms of another pathogen infection, leading to more severe disease (Bains and Parkash, 1984; Omar et al., 1986; Meyer and Pataky, 2010; Tollenaere et al., 2017; Philosoph et al., 2018). For example, the tobamovirus cucumber green mottle mosaic virus (CGMMV) has been shown to increase the severity of *Pythium* wilting in cucumber under various environmental conditions (Philosoph et al., 2018; Philosoph et al., 2019). Other outcomes of co-infection with viruses and phytopathogens have been reported, with viral infection reducing disease susceptibility in certain cases (Latch and Potter, 1977; Varughese and Griffiths, 1983). However the molecular pathways determining these interactions remain unclear.

*Botrytis cinerea* (*B. cinerea*, Bc) is the causative agent of gray mold disease (Williamson et al., 2007). *B. cinerea* infects upwards of 1400 plant hosts, many of them crop plants, and causes severe damage in greenhouses and post-harvest storage and transport (Williamson et al., 2007; Elad et al., 2016; Fillinger and Elad, 2016). *Xanthomonas euvesicatora* (*Xcv*) causes bacterial leaf spot (BLS) on peppers and tomatoes. BLS severely reduces plant productivity and yield (Jones et al., 2004). Since *B. cinerea* and *X. euvesicatoria* are important pathogens in tomato agricultural cropping (Moss et al., 2007; Fillinger and Elad, 2016), and since the tobamovirus ToBRFV has become widespread in worldwide tomato agriculture (Zhang et al., 2022a), we were interested to examine the impact of the interaction between *B. cinerea*/*X. euvesicatoria* and tobamoviruses on tomato disease response.

Different pathogens can activate common or different pathways in their plant host. The nature of the defense pathways activated during infection with more than one pathogen will likely influence the eventual resistance or susceptibility to each pathogen. *B. cinerea*, primarily a necrotrophic plant pathogen, is known to activate Jasmonic acid (JA) pathway defenses (Rowe et al., 2010; Mehari et al., 2015; Fillinger and Elad, 2016). Mutant plants with impaired JA content or sensing are notoriously sensitive to *B. cinerea* infection (AbuQamar et al., 2008; Gupta et al., 2021a; Gupta et al., 2022). *B. cinerea* was also demonstrated to activate SA-dependent pathways *in planta*, promoting pathogenesis (El Oirdi et al., 2011; Rossi et al., 2011). Tobamoviruses are known to activate a different plant defense response pathway, the salicylic acid (SA) pathway (White, 1979; Gaffney et al., 1993; Murphy and Carr, 2002). While JA confers defense against necrotrophic pathogens, SA is largely thought to confer protection against biotrophic pathogens (Yang et al., 2015). The competitive crosstalk between the JA and SA pathways determines the precise defense response to the specific attacker (Thaler et al., 2012; Caarls et al., 2015; Aerts et al., 2021). In this work, we found that infection with the tobamoviruses ToMV or ToBRFV resulted in increased *B. cinerea* incited disease, likely through increases in endogenous SA and activation of the SA pathway. Our results suggest that manipulation of plant defense pathways by viruses may lead to unexpected outcomes, which include increased susceptibility to additional pathogens. Given that the increased pathogen susceptibility may ultimately cause more damage than the original virus, future management strategies for plant viruses should take into account both the primary damage caused by viruses, and the secondary effect of plant viruses on susceptibility to additional pathogens such as *B. cinerea*.

## Materials and methods

### Plant materials and growth conditions

*Solanum lycopersicum* cv Moneymaker (“Mm”) and the transgenic SA degrading *NahG* line (Gupta et al., 2021b) were grown from seeds in soil (Green Mix 443; Even‐Ari, Ashdod, Israel) in a growth chamber, under long day conditions (16 hr:8 hr, light:dark) at 24°C.

### Tobamovirus infection

Tobamovirus infection was performed as described (Vaisman et al., 2022). Briefly, 5 gr of symptomatic leaves from plants infected with either ToMV (accession no. NC_002692.1) or ToBRFV (accession no. KX619418.1) were crushed using mortar and pestle and supplemented with carborundum powder and 50 ml of phosphate buffer (0.01 M NaH_2_PO_4_) 0.01 M. Inoculation was done on cotyledons of two-week-old tomato seedlings by gently rubbing them with the crushed leaf solution.

### *B. cinerea* inoculation using mycelial plugs

*B. cinerea* (isolate Bc16) was cultured on potato dextrose agar (PDA) (Difco Lab) and incubated at 22°C for 3-5 days. Leaves number 5-6 from 5-week-old tomato plants (three weeks after *tobamovirus* inoculation) were excised and placed in humid chambers. Mycelial plugs (5 mm diameter) from a 3-day-old culture were taken using a cork-borer from colony margins, and placed mycelial side down on the adaxial surface of each leaflet. Inoculated leaves were kept in a humid growth chamber at 21°C for three days. Assay was repeated four independent times.

### *Xanthomonas euvesicatoria* inoculation by infiltration

*X. euvesicatoria* bacterial cultures were grown in LB medium containing 100 mg/L of rifampicin and 300 mg/L of streptomycin, overnight at 28°C (O’Donnell et al., 2001). Log phase bacterial cultures were harvested and re-suspended in 10 mM MgCl_2_, at a final concentration of 10^4^ CFU/mL (OD_600 =_ 0.0002). The fourth leaf of 5-week-old tomato plants (3 weeks after tobamovirus inoculation) was infiltrated with the bacterial suspensions using a needless syringe. To determine CFU, five days after injection, three leaf discs of 0.9 cm diameter were sampled from the second left-hand lateral leaflet from the inoculated leaf of at least five plants, and ground in 1 mL of 10 mM MgCl_2_. CFU were determined by plating serial dilutions, and counting the resultant colonies (Lund et al., 1998). 10 mM MgCl_2_ without pathogen inoculation was used as a negative control. Water-soaked lesions were measured 10 days post inoculation (dpi) as previously described (Teper et al., 2018). Assay was repeated three independent times.

### Immunity assays

All immunity assays were conducted on mature leaves from ToMV- and ToBRFV- inoculated plants as well as non-inoculated control plants.

ROS measurement was performed as previously described (Leibman-Markus et al., 2017). Leaf disks 0.5 cm in diameter were taken from leaves 5-6 of 4-5-week-old plants. Disks were floated in a white 96-well plate (SPL Life Sciences, Korea) containing 250 μL distilled water for 4–6 h at room temperature. After incubation, water was removed and a ROS measurement reaction containing either 1 μg/mL EIX (Anand et al., 2021), 1 μM flg-22 (PhytoTech labs), or water (mock) was added. Light emission was immediately measured using a luminometer (GloMax^®^ Discover, Promega, USA).

Conductivity assays were performed according to Leibman-Markus et al. (2017). 0.9 cm diameter leaf discs were harvested from leaves 5-6 of 4-5-week-old plants and washed with distilled water for 3 hours in a 50 mL tube. For each sample, five discs were placed in a 10-flask with 1 ml of distilled water, for 48 hours with agitation. After incubation, 1.5 mL of distilled water were added to each sample, and conductivity was measured using a conductivity meter (AZ® Multiparameter pH/Mv/Cond./Temp Meter 86505, Taiwan).

### SA treatments

To simulate virus-induced SA production, SA solutions of 1, 2 or 5 mM in distilled water were sprayed to drip onto aerial plant parts. Plants were given four treatments every 3-4 days over a 2-week period, after which disease susceptibility was assessed.

### Gene expression and RT-qPCR

For RT-qPCR analyses, total RNA was prepared using Tri-reagent (Sigma-Aldrich) according to the manufacturer’s instructions. Three μg of RNA were converted to first-strand cDNA using reverse transcriptase (Promega, Madison, WI) and oligo-d(T) primers. RT-qPCR was performed according to the Power SYBR Green Master Mix protocol (Life Technologies, Rockville, MD), using a StepOnePlus machine (Thermo Fisher, Waltham, MA, United States). **Supplementary Table S1** lists the primers used in this study and primer pair efficiencies. Systemic viral infection was confirmed by quantifying the expression of ToBRFV and ToMV coat protein genes relative to the housekeeping gene TIP41 (Hak and Spiegelman, 2021; Kravchik et al., 2022). Gene expression was normalized to the geometric mean of the expression of two housekeeping genes: Cyclophilin (CYP) (Solyc01g111170), a housekeeping gene previously used in qPCR experiments in tomato (Mascia et al., 2010), and the ribosomal protein L8 (RPL8) (Solyc10g006580), which was previously used for the normalization of gene expression in pathogen assays in tomato (Meller Harel et al., 2014; Gupta et al., 2021a). Relative expression quantification was done using the copy number method for gene expression (D’haene et al., 2010).

### SA measurements in tomato leaf tissues

Tissue for SA content quantification was harvested 2 weeks after tobamovirus infection. Each sample consisted of 5 independent biological replicates. SA was measured as described (Shaya et al., 2019; Gupta et al., 2020). Tissue was harvested, frozen, and immediately ground to a fine powder. One gram of powder was suspended in two vials containing 1 mL extraction solvent (ES) (79% isopropanol: 20% methanol: 1% acetic acid) with the addition of 20 ng of labeled internal standard (Sigma). The samples were incubated at 4°C for 1 h with rapid shaking and subsequently centrifuged at 14,000g for 15 min. The supernatant was collected and 500 µL of ES was added to the pellet, with the extraction steps being repeated an additional two times. Combined extracts were then evaporated using speed-vac at room temperature. The dried samples were dissolved in 200 μl of 50% methanol, and filtered through a 0.22 μm cellulose syringe filter. 5–10 μl of each methanol-dissolved sample was injected for each analysis. LC–MS-MS analyses were conducted using a UPLC-TripleQuadrupoleMS (WatersXevo TQMS). Quantification was performed using isotope-labeled internal standards.

### Statistical analyses

Data sets were analyzed for normality using the Shapiro-Wilk test. For normally distributed samples, differences between two groups were analyzed for statistical significance using a two tailed t-test, with Welch’s correction for samples with unequal variances, where appropriate. Differences among three groups or more were analyzed for statistical significance using one-way analysis of variance (ANOVA), or Welch’s ANOVA (when samples within the group were determined to have unequal variances). When a significant result for a group in an ANOVA was returned, significance in differences between the means of different samples in the group were assessed using Tukey’s *post-hoc* test (equal variances), or Dunnett’s *post-hoc* test (unequal variances). For samples not normally distributed, differences between two groups were analyzed for statistical significance using Mann-Whitney’s U test, and differences among three groups or more were analyzed for statistical significance using Kruskal-Wallis ANOVA, with Dunn’s *post-hoc* test. All statistical analyses were conducted using Prism9™. All experiments were conducted in at least three biologically independent repeats. The number of replicates and the statistical tests used in each case are indicated for each experiment in the corresponding figure legend.

## Results

### Infection with tobamoviruses influences susceptibility to additional pathogens

**Figure 1:**
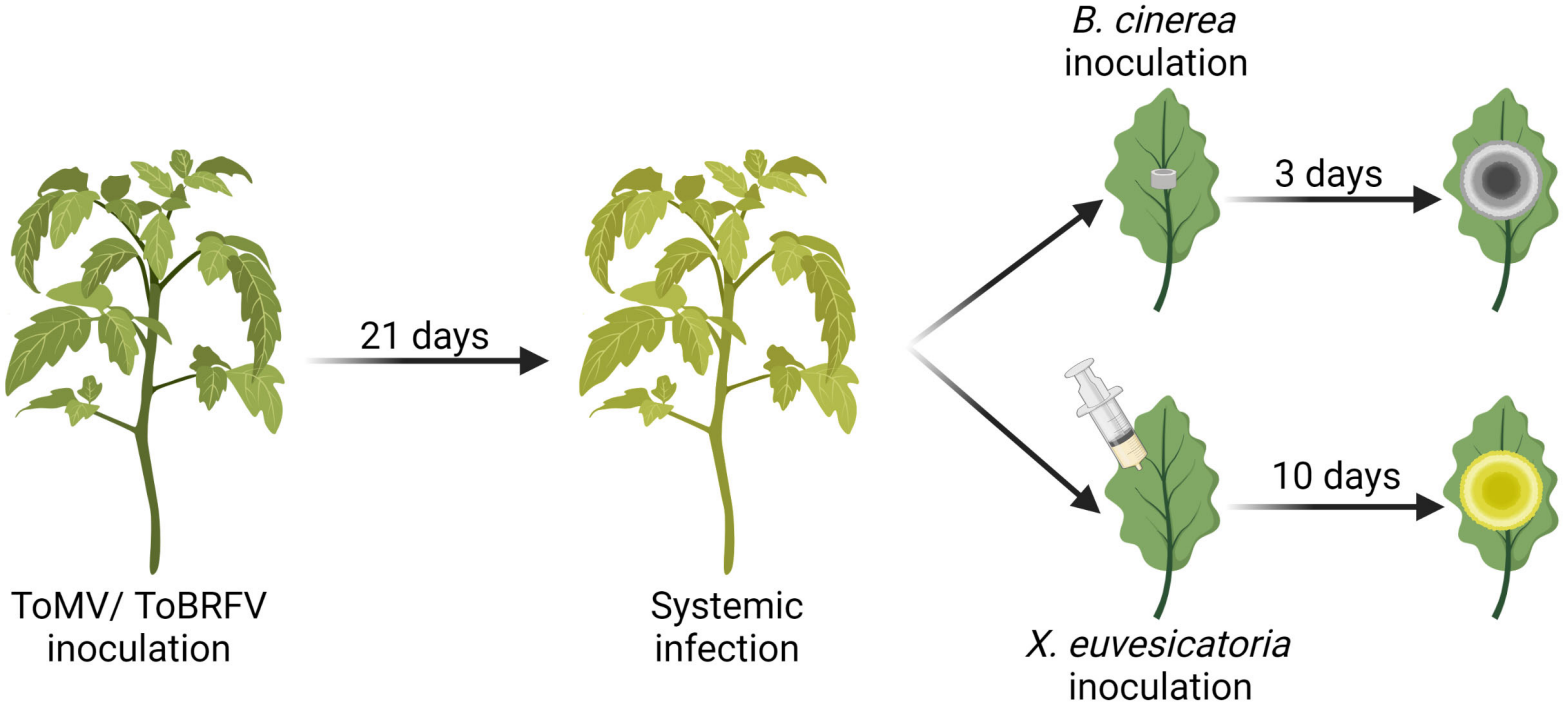
Schematic illustration of the double infection assays performed in this study. *Solanum lycopersicum* cv. Moneymaker plants were inoculated with ToMV or ToBRFV at 2 weeks of age. Three weeks after inoculation, tobamovirus infection was confirmed by qRT-PCR and by the appearance of visual viral symptoms. At this point, fully expanded mature leaves (number 4-6) were challenged with *B. cinerea* mycelia plugs or infiltrated with *X. euvesicatoria* culture (OD_600 =_ 0.0002). Symptoms of *B. cinerea* and *X. euvesicatoria* were documented 3 and 10 days after inoculation, respectively. Illustration created using Biorender.com.

**Figure 2:**
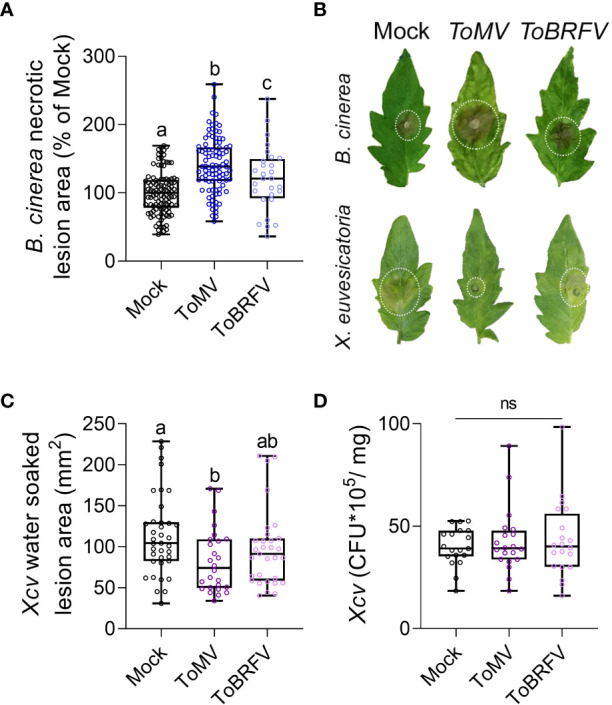
Tobamovirus infection modulates pathogen resistance. *S. lycopersicum* cv. Moneymaker plants were infected with ToMV or ToBRFV at 2 weeks of age. 3 weeks after viral inoculation, plants were challenged with **(B)** *cinerea* 3-day-old mycelia **(A, B)** or *X. euvesicatoria* (*Xcv*, OD_600 =_ 0.0002, **B-D**) on leaves 4-5. **(A)** Necrotic lesion area was quantified 3 days after *B. cinerea* inoculation. Experiment was repeated 4 independent times. **(B)** Representative images of mock, ToMV- and ToBRFV- infected plants inoculated with *B. cinerea* or *X. euvesicatoria*. **(C)** Water-soaked lesion area was quantified 5 days after *X. euvesicatoria* inoculation. Experiment was repeated 3 independent times. **(D)** Leaf tissue samples from 5 individual plants per experiment was ground in MgCl_2_ and CFU were counted from plated serial dilutions 5 days after *X. euvesicatoria* inoculation. Experiment was repeated 3 independent times. Boxplots represent inner quartile ranges (box), outer quartile ranges (whiskers), median (line in box), all points shown. Different letters indicate statistically significant differences between samples in one way ANOVA with Tukey’s *post hoc* test **(A, D)**, or in Welch’s ANOVA with Dunnett’s *post hoc* test **(C)**. A: N>30, p<0.038. C: N>30, p<0.018. D: N=20. ns, not significant.

Increased disease susceptibility following virus infection has been reported in several plant hosts (Latch and Potter, 1977; Bains and Parkash, 1984; Omar et al., 1986; Meyer and Pataky, 2010; Philosoph et al., 2018). Although this phenomenon has been known for many years, the molecular mechanism underlying it is still unclear. To explore the effect of tobamoviruses on disease responses in tomato, we used the necrotrophic fungal pathogen *Botrytis cinerea*, and the hemibiotrophic bacterial pathogen *Xanthomonas euvesicatoria*. The experimental procedure is depicted in **Figure 1**. Two-week-old *Solanum lycopersicum* cv Moneymaker (Mm) plants were inoculated with either ToMV or ToBRFV. Plants were grown for 3 weeks after inoculation, and systemic infection was confirmed by the appearance of systemic viral symptoms and by RT-qPCR on systemic leaf tissue (**Figure 1**, **Supplementary Figure S1**). Leaflets of mature leaves were then challenged with either *B. cinerea* or *X. euvesicatoria*, and the response to each pathogen was measured by the lesion size at 3 (*B. cinerea*) or 10 (*X. euvesicatoria*) days post inoculation (dpi). Infection with ToMV and ToBRFV increased the lesion generated by *B. cinerea* (**Figures 2A, B**). The increased disease severity was greater with ToMV as compared to ToBRFV (**Figure 2A, B**). Infection with ToMV reduced the disease symptoms caused by *X. euvesicatoria* (**Figures 2B, C**), though CFU numbers were unaffected (**Figure 2D**). These results suggest that while ToMV infection did not have an effect on *X. euvesicatoria* growth, it did affect the host response to this pathogen. Susceptibility to *X. euvesicatoria* was not significantly affected by ToBRFV infection (**Figures 2C, D**).

### Infection with tobamoviruses affects cellular immunity and defense gene expression

**Figure 3:**
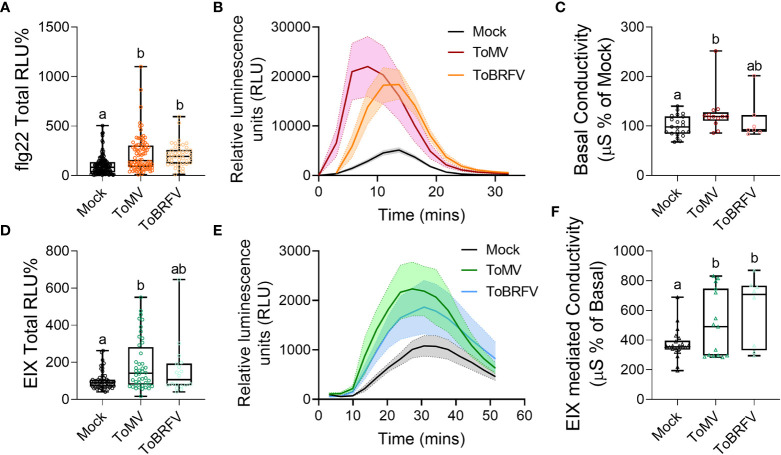
Tobamovirus infection modulates plant immunity. *S. lycopersicum* cv. Moneymaker plants were infected with ToMV or ToBRFV at 2 weeks of age. Three weeks after viral inoculation, plants were challenged with the immunity elicitors flg22 **(A, B)** or EIX **(D-F)**, or wounded **(C)**. Reactive oxygen species (ROS) burst in response to the bacterial elicitor flg-22 **(A, B)** or the fungal elicitor EIX **(D, E)**, and electrolyte leakage in response to wounding **(C)** or EIX **(F)** were quantified. **(A, D)**: Total ROS produced, expressed as Relative luminescence units (RLU), in % of Mock. **(B, E)**: Kinetics of the ROS burst. Line indicates mean of each time point, shaded area depicts SE. **(C)**: Basal conductivity as a result of wounding. F: EIX-mediated conductivity. At least three independent experiments were conducted in all cases, **(A, B)**: N≥45, **(D, E)**: N≥18, **(C)**: N≥7, **(F)**: N≥8. **(A, C, D, F)**: Boxplots represent inner quartile ranges (box), outer quartile ranges (whiskers), median (line in box), all points shown. Different letters indicate statistically significant differences between samples in Welch’s ANOVA with Dunnett’s *post hoc* test **(A, C, D)**, or in one way ANOVA with Tukey’s *post hoc* test **(F)**. A: p<0.0001; C: p<0.023; D: p<0.0002; F: p<0.046.

To examine the underlying factors modulating disease susceptibility following virus infection, we next assayed immune system activation and defense gene expression following infection with ToMV and ToBRFV. Both viruses increased ROS production in response to elicitation with the bacterial flagellin peptide flg-22 (Pizarro et al., 2020) (**Figures 3A, B**), while pre-infection by ToMV also promoted ROS production in response to the fungal elicitor EIX (ethylene-inducing xylanase (Anand et al., 2021), **Figures 3D, E**). ToMV caused an increase in basal conductivity (**Figure 3C**), and both viruses increased conductivity in response to EIX treatment (**Figure 3F**). These results suggest that tobamovirus infection alters the plant immune response to pathogen elicitors.

**Figure 4:**
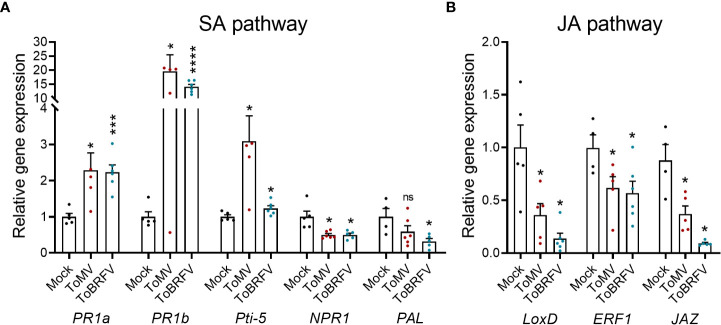
Tobamovirus infection alters SA and JA pathway gene expression. *S. lycopersicum* cv. Moneymaker plants were infected with ToMV or ToBRFV at 2 weeks of age. Three weeks after viral inoculation, cDNA was prepared from RNA extracted from the fourth leaf of 3 individual plants. SA **(A)** and JA **(B)** pathway gene expression was assayed by RT-qPCR. Experiment was repeated twice, N=6. Bars represent mean ± SE, all points shown. Asterisks indicate statistically significant differences in gene expression in virus-infected plants as compared with mock plants, in Welch’s *t-rom* comparing each gene. *p<0.05, ***p<0.001, ****p<0.0001, ns, not significant.

To gain insights into the immune pathways altered during viral infection, defense gene transcripts were quantified using RT-qPCR (**Figure 4**). The SA pathway genes *pathogenesis related 1a* (*PR1a*), *PR1b*, and *pathogen-inducible 5* (*Pti-5*) (Yalpani et al., 1991; Gu et al., 2002), were up-regulated by both ToMV and ToBRFV-infected plants (**Figure 4A**). In marked contrast, the SA receptor *nonexpressor of pathogenesis-related genes 1* (*NPR1*) and the SA biosynthetic pathway gene *phenylalanine lyase* (*PAL*) (Cao et al., 1997; Lin et al., 2004; Kim and Hwang, 2014) were down-regulated following tobamovirus infection, possibly due to a negative feedback mechanism curbing the SA response (**Figure 4A**). In addition, the JA pathway genes *lipoxygenase D* (*LoxD*), *ethylene response factor 1 (ERF1*), and *jasmonate zim domain protein* (*JAZ*) (Ishiga et al., 2013; Meller Harel et al., 2014; Gupta et al., 2022) were all repressed by infection with tobamoviruses (**Figure 4B**). These results suggest that tobamovirus infection upregulates the SA response pathway while suppressing the JA response pathway.

### Alterations in disease susceptibility following tobamovirus infection could result from increases in SA levels

**Figure 5:**
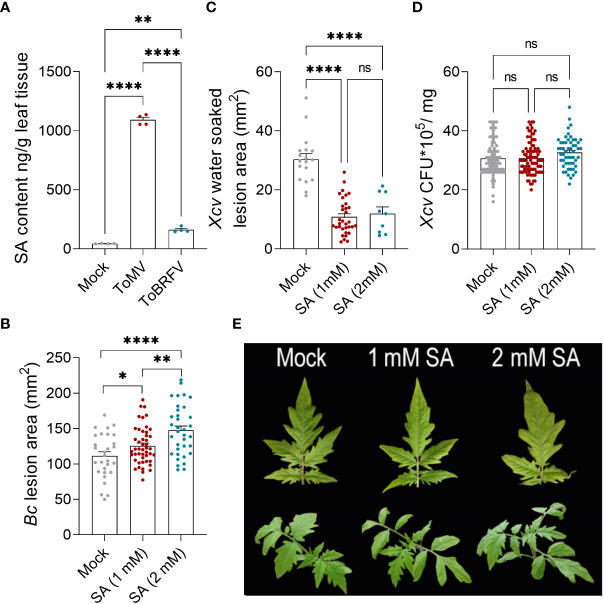
Tobamovirus infection alters pathogen susceptibility through the SA pathway. **(A)** *S. lycopersicum* cv. Moneymaker plants were infected with ToMV or ToBRFV at 2 weeks of age. Three weeks after viral inoculation, SA was quantified by LC-MS-MS in the fourth leaf of four individual plants. **(B-D)** *S. lycopersicum* cv. Moneymaker plants were treated with indicated concentrations of SA by foliar spray for 2 weeks (4 treatments in total). Three days after the last treatment, plants were challenged with *B. cinerea* 3-day-old mycelia **(B)** or *X. euvesicatoria* (*Xcv*, OD_600 =_ 0.0002, **(C, D)** on leaves 4-5. **(B)** Necrotic lesion area was quantified 3 days after *B. cinerea* inoculation. Experiment was repeated three independent times. **(C)** Water-soaked lesion area was quantified 10 days after *X. euvesicatoria* inoculation. Experiment was repeated four independent times. **(D)** Bacterial CFU were quantified 5 days after *X. euvesicatoria* inoculation. Experiment was repeated four independent times. **(E)** Representative images of SA treated plants after four treatments. Bars represent mean ± SE, all points shown. Asterisks indicate statistically significant differences between indicated samples in Welch’s ANOVA with Dunnett’s *post hoc* test. A: N=4, p<0.005. B: N>30, p<0.036. C: N>9, p<0.0001. D: N>50, p>0.1. *p<0.05, **p<0.01, ****p<0.0001, ns, not significant.

Tobamovirus infection is known to activate SA production in plant cells (Malamy et al., 1990; Conti et al., 2017). Increased SA biosynthesis may therefore explain the activation of SA response pathways due to tobamovirus infection. We directly compared the levels of SA elicited in tomato leaves following infection with ToMV and ToBRFV. While infection with both of these viruses enhanced SA production, ToMV increased leaf SA levels about 25-fold, while ToBRFV increased leaf SA levels about 4-fold (**Figure 5A**).

Elicitation of SA production and alterations in disease response and cellular immunity were more pronounced with ToMV infection (**Figures 2**-**4**). Since SA levels have been shown to affect disease responses (Liu et al., 2016; Di et al., 2017; Betsuyaku et al., 2018; Li et al., 2019), we investigated whether treating tomato plants with exogenous SA at similar levels and durations elicited by 2-to-3-week tobamovirus infections could affect disease responses in a similar manner to viral infection. Tomato plants were treated with 1 mM (similar to levels measured following ToBRFV infection) or 2 mM SA. We used 2 mM to simulate ToMV infection, though it is much lower than the endogenous levels of SA elicited by ToMV, which corresponds to about 8 mM, because exogenous treatment with 5 mM SA was toxic to tomato plants (**Supplementary Figure S2**). SA treatments at both concentrations resulted in increased severity of gray mold disease, and moderation of *X. euvesicatoria-*elicited symptoms (**Figures 5B, C**). Bacterial load remained unaffected in all treatments (**Figure 5D**). These results were similar to the effect of tobamovirus infection on the disease caused by both pathogens (**Figure 2**).

**Figure 6:**
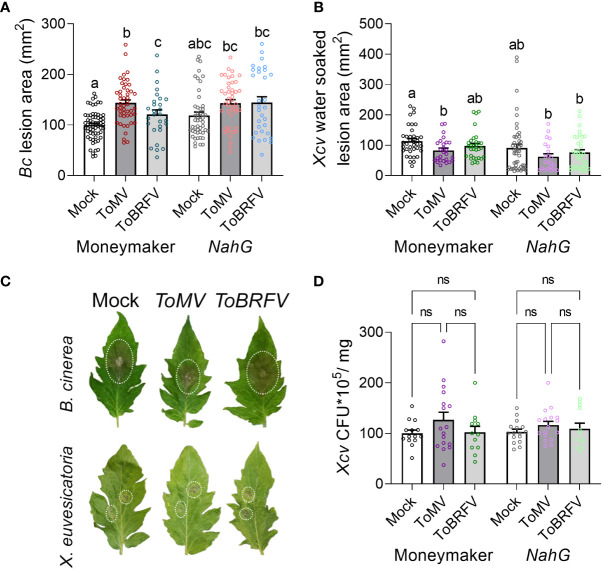
Tobamovirus infection requires SA to modulate pathogen resistance. *S. lycopersicum* cv. Moneymaker control plants or SA-degrading “*NahG*” transgenic plants were infected with ToMV or ToBRFV at 2 weeks of age. Three weeks after viral inoculation, plants were challenged with *B. cinerea* 3-day-old mycelia **(A, C)** or *X. euvesicatoria* (*Xcv*, OD_600 =_ 0.0002, **(B-D)**. **(A)** Necrotic lesion area was quantified 3 days after *B. cinerea* inoculation. Experiment was repeated 4 independent times. **(B)** Water-soaked lesion area, and **(D)** bacterial CFU, were quantified 10 and 5 days after *X. euvesicatoria* inoculation, respectively. Experiment was repeated 3 independent times. **(C)** Representative images of mock, ToMV- and ToBRFV- infected *NahG* plants inoculated with *B. cinerea* or *X. euvesicatoria*. Bars represent mean ± SE, all points shown. Different letters indicate statistically significant differences between samples in one way ANOVA with Tukey’s *post hoc* test **(A)**, or in Welch’s ANOVA with Dunnett’s *post hoc* test **(B, D)**. A: N>28, p<0.011. B: N>22, p<0.037. D: N>12, p>0.52. ns, not significant.

To further explore the role of the increase in SA in alterations observed to disease susceptibility following tobamovirus infection, we examined the effect of tobamovirus infection on disease susceptibility in the SA-deficient *NahG* transgenic line, which lacks endogenous SA (Mehari et al., 2015; Gupta and Bar, 2020). Interestingly, while tobamovirus infection increased the *B. cinerea* lesion size in control plants (cv. Moneymaker), it did not have an effect in the *NahG* transgenic tomato line (**Figures 6A, C** **)**. In addition, while ToMV infection reduced the *X. euvesicatoria* water soaked lesion area in control plants, it had no effect over lesion size in *NahG* plants (**Figures 6B, C**). Collectively, these results suggest that tobamovirus infection alters the plant response to *B. cinerea* and *X. euvesicatoria* in an SA-dependent manner.

## Discussion

Plants often encounter multiple pathogens in their environment (Abdullah et al., 2017). In addition to possible interactions between two or more pathogenic microorganisms on or within the plant, the interaction of each pathogen with the plant host can shape the hosts’ subsequent response to additional pathogens. Interactions between plant-infecting microorganisms can be symbiotic, synergistic, or antagonistic, either directly, or through the plant host (Abdullah et al., 2017). In our work, we found that the tobamoviruses ToMV and ToBRFV cause aggravation of the disease caused by the necrotrophic fungal pathogen *B. cinerea*, and moderation of the disease caused by the hemibiotrophic bacterial pathogen *X. euvesicatoria* (**Figure 2**). These interactions are likely generated by the response of the tomato host, through increases in SA levels (**Figure 5**), which aggravated the symptoms caused by the necrotroph *B. cinera* and attenuated the symptoms of the biotroph *X. euvesicatoria*. Comparison between the two studied tobamoviruses demonstrates that ToMV promoted greater increases in SA levels, stronger susceptibility to *B. cinerea*, and resistance to X*. euvesicatoria*, while ToBRFV promoted milder increases in SA levels, weaker susceptibility to *B. cinerea*, and no resistance to X*. euvesicatoria* (**Figures 2**, **5**). Exogenous SA treatment also resulted in susceptibility to *B. cinerea* elicited disease and resistance to X*. euvesicatoria* elicited disease (**Figure 5**), suggesting that these differential responses observed in virus-infected plants are due to increased SA levels. In addition, *NahG* plants lacking endogenous SA were not able to respond to tobamovirus infection with increases in *B. cinerea* susceptibility or *X. euvesicatoria* resistance (**Figure 6**). Taken together, these findings support the hypothesis that the effects of viral infection on pathogen susceptibility observed in our system indeed involve SA-regulated pathways. It is important to note that when studying dual infections, the relative timing of both infections may alter the experimental outcome. In our system, the second pathogen infection occurred after systemic viral infection had occurred (21 dpi) and the virus was fully established in its host. It is possible that co-infection at earlier stages, for example during the first days of infection when the virus had not yet moved out of the infected leaf, may yield different results.

SA promotes resistance to many plant pathogens, including bacteria, fungi and viruses (Peng et al., 2021), and is required for plant immunity to (hemi)biotrophic pathogens. SA-mediated immunity has typically been described as occurring *via* activation of the SAR pathway (Fu and Dong, 2013; Di et al., 2017). Interestingly, early investigations of plant response to the tobamovirus TMV uncovered that SA promotes plant resistance to TMV (White, 1979). Plants which are unable to accumulate SA are highly susceptible to TMV (Gaffney et al., 1993), as SA reduces viral accumulation and cell-to-cell movement (Murphy and Carr, 2002). Tobamovirus infected plants were previously shown to accumulate high levels of SA (Malamy et al., 1990; Conti et al., 2017). We also observed hyper-accumulation of endogenous SA contents in ToMV- and ToBRFV- infected plants (**Figure 5**). Previously, we demonstrated that the ability of ToBRFV to break *Tm-2^2^* resistance is linked to its moderated mobility (Hak and Spiegelman, 2021; Hak et al., 2023), suggesting that ToMV spreads faster than ToBRFV in a tobamovirus*-*susceptible background. However, since there is currently no genetic resistance to ToBRFV in tomato, it poses a much greater agricultural challenge. Interestingly, our current work demonstrates that the reduced movement of ToBRFV (Hak and Spiegelman, 2021) correlated with a lower ability to promote increases in plant SA, as compared with ToMV (**Figure 5**). Thus, it would seem that systemic plant movement is correlated with the plants’ SA production, and the plant activates its SA pathways at a level commensurate with the infectivity of the virus attacking it.

Elevated SA levels are known to promote susceptibility to necrotrophic pathogens, including *B. cinerea* (Mengiste, 2012). Exogenous SA treatment, and endogenous SA accumulation following biotrophic pathogen infection, were both previously demonstrated to increase susceptibility to the necrotrophic fungal pathogen *A. brassicicola* in *Arabidopsis* (Spoel et al., 2007), due to inhibition of JA dependent defenses by SA. Loss of *Arabidopsis thaliana* BOTRYTIS-INDUCED KINASE1 (BIK1) enhanced *B. cinerea* susceptibility in an SA-dependent manner (Veronese et al., 2006), and *B. cinerea* is known to activate the SA pathway in host plants (El Oirdi et al., 2011; Rossi et al., 2011). Suppression of the tobacco mitogen-activated protein kinase phosphatase (NtMKP1) negatively regulated SA-mediated tissue necrosis and as a result enhanced resistance to *B. cinerea* (Oka et al., 2013). Furthermore, viral infection has been demonstrated to promote susceptibility to several necrotrophic pathogens, including *B. cinerea* (Omar et al., 1986; Meyer and Pataky, 2010; Philosoph et al., 2018). Our results suggest that the tobamovirus-mediated hyper-accumulation of SA may increase susceptibility to *B. cinerea* elicited disease in a similar manner.

While SA is considered to promote resistance against viruses, in some cases, SA fails to suppress viral infection (Murphy et al., 2020). In fact, several viruses promote SA biosynthesis and signaling during systemic infection (Krečič-Stres et al., 2005; Zhou et al., 2014; Shaw et al., 2019). It is therefore possible that while SA accumulates during viral infection, viruses suppress specific elements downstream to SA to successfully infect the plant. For example, the tobamovirus movement protein suppresses callose deposition near plasmodesmata (Guenoune-Gelbart et al., 2008), an SA- dependent process (Wang et al., 2013). The coat protein suppresses the expression of several SA-responsive genes to allow systemic transport of the virus (Conti et al., 2012; Venturuzzi et al., 2021). Other SA-mediated responses, which are not suppressed by the virus, would likely remain activated (Laird et al., 2013; Yang et al., 2016). Alternatively, the upregulation of SA could reflect a failed defense response, which is initiated after the virus is already established. This type of dysfunctional immune response may cause deleterious effects, including enhanced disease symptoms. Indeed, several studies have shown that strong viral symptoms are often associated with upregulation of the SA-responsive genes and metabolites (Diaz-Vivancos et al., 2006; Hernández et al., 2006; Niehl et al., 2006; Kogovšek et al., 2010; Shang et al., 2010). These observations suggest that during viral infection, the upregulation of several SA-mediated pathway elements may enhance the disease symptoms rather than protect plants against the virus. We propose that another effect of this immune activation may be the alteration of plant response to other pathogens.

Reportedly, SAR depends on the SA pathway, while ISR relies on JA/ET pathways. While SA is antagonistic to JA in defense signaling (Pieterse et al., 2009; Pieterse et al., 2012), there are cases where SA and JA were reported to positively interact (Ferrari et al., 2003; De Vleesschauwer et al., 2013; Liu et al., 2016; Ullah et al., 2022). Dependence of ISR on SA signaling has been reported as well (Martínez-Medina et al., 2013). Distinctions between ISR and SAR are not clear cut in tomato, and overlap between the two was reported (Liu et al., 2016; Betsuyaku et al., 2018). Thus, while plant defense against *B. cinerea* requires the JA-mediated pathway (AbuQamar et al., 2008), and plant defense against viruses involves the SA-mediated pathway (White, 1979; Malamy et al., 1990), we found that the plants response to tobamoviruses and *B. cinerea* operates in the same direction, with virus-infected plants becoming more susceptible to *B. cinerea*, in a SA-dependent manner (**Figures 2**; **5** **-** **6**). This is likely due to antagonism between the high levels of SA elicited by the viral infection (**Figure 5**), and JA required to promote plant defenses against *B. cinerea*.

It should be noted that the SA-mediated effects on *B. cinerea* pathogenesis may also depend on the specific concentration of SA. For example, low levels of the SA analog acibenzolar-S-methyl promoted resistance to *B. cinerea* in tomato (Leibman-Markus et al., 2023), and priming SA-mediated defenses was also found to induce *B. cinerea* resistance (Audenaert et al., 2002; Gupta et al., 2020; Gupta et al., 2022; Marash et al., 2022). Here, ToMV infection resulted in SA levels of approximately 1000 ng/gr, which is equivalent to ~8mM. However, exogenous application of 5 mM resulted in phytotoxicity. Interestingly, we found that during tobamovirus infection, the levels of the SA receptor NPR1 are down-regulated (**Figure 4A**). This negative feedback mechanism may curb the SA response even when SA levels are exceedingly high, and may explain how the plant tolerates high endogenous doses of SA during infection. Nevertheless, the excess levels of SA may still create conditions that are favorable for necrotrophs by promoting cell death.

Our results lead to the conclusion that in certain cases, co-infection with biotrophs and necrotrophs, or SA-dependent and JA-dependent pathogens, could be particularly devastating, because the strong plant response to one type of pathogen makes the host unable to properly respond to another type of pathogen. This could have implications both in co-infection research and in agricultural cultivation. Different pathogens can be inexplicably aggressive in particular growing seasons, and it is plausible that one of the underlying causes of this might be strong activation of one pathway of the plant immune or stress response system, leaving the plant unable to respond to subsequent challenge. However, this is only relevant to a sub-set of phytopathogens and plant host systems, as viral infection was also reported to increase susceptibility to biotrophic pathogens (Latch and Potter, 1977; Bains and Parkash, 1984), and SA and JA are known to have both positive and negative interactions in plant defense (Takahashi et al., 2004; Li et al., 2019; Vlot et al., 2021; Ullah et al., 2022). These findings need to be taken into consideration when designing induced resistance protocols to mitigate plant viruses. While SAR-inducing compounds, such as acibenzolar-s-methyl were found to be effective against some viruses (Palukaitis et al., 2017), they may have a negative effect on the disease response to other pathogens, when co-infection occurs. The use of specific resistance inducers should therefore be tailored according to the needs of the specific crop, taking into account the additional potential pathogens within each specific cropping environment.

Based on our results it seems that tobamoviruses, which pose a substantial economic problem in worldwide agriculture (Zhang et al., 2022a), can also increase crop losses by making plants more susceptible to other classes of pathogens. This could be possibly mitigated by the fact that the same virus or viruses can generate some resistance to other classes of pathogens. Thus, the balance between pathogen resistance and pathogen susceptibility in a particular growing season, when dealing with tobamovirus infected plants, will depend on the prevalence of particular pathogens in the cropping environment. Our results indicate that resistance or susceptibility to different classes of pathogens in tobamovirus-infected plants are primarily a result of substantial changes to the SA pathway. Further research is needed to elucidate the relationships between different defense pathways during tobamovirus infection, as well as the roles of SA in *B. cinerea* pathogenesis and its interactions with the plant host.

## Data availability statement

The original contributions presented in the study are included in the article/**Supplementary Material**. Further inquiries can be directed to the corresponding authors.

## Author contributions

ZS and MB: conceptualization. ML-M, RG, DW, ZS and MB: methodology. ML-M, RG, and DW: investigation. ML-M, RG, DW, ZS and MB: analysis. ZS and MB: writing – original draft. ML-M, RG, DW, ZS and MB: writing – review and editing. ZS and MB: funding acquisition. All authors contributed to the article and approved the submitted version.

## Supplementary material

Supplementary Figure 1 Assessment of tobamovirus infection.

Supplementary Figure 2 5mM SA is phytotoxic.

Supplementary Table 1 Primer pairs used in qRT-PCR.
