# Supplementary material for: Tobamovirus infection aggravates gray mold disease caused by Botrytis cinerea by manipulating the salicylic acid pathway in tomato
Source: Front Plant Sci. 2023 Jun 12;14:1196456. doi: 10.3389/fpls.2023.1196456 (PMC10291333; doi:10.3389/fpls.2023.1196456)
Supplement: Supplementary Figure 1 — Assessment of tobamovirus infection. [file DataSheet_1.pdf]

Supplemental materials

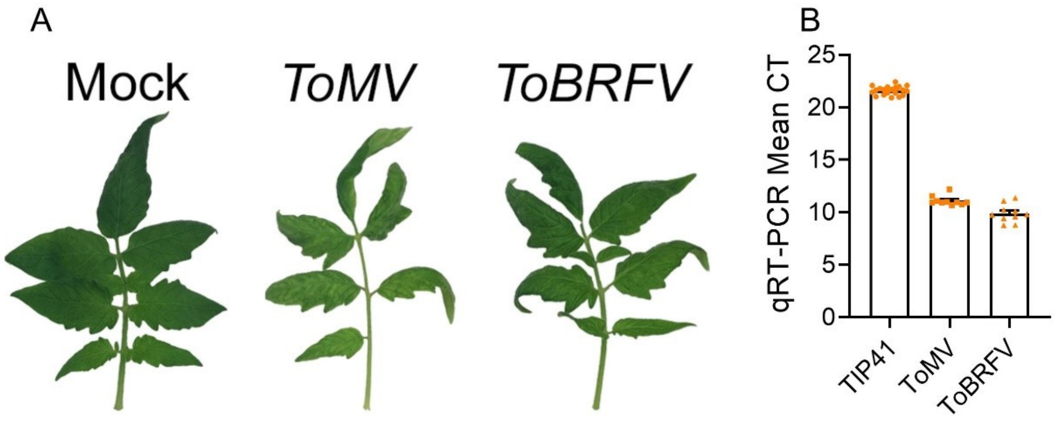

Figure S1: Assessment of tobamovirus infection.

S. lycopersicum cv. Moneymaker plants were infected with ToMV or ToBRFV at 2 weeks of age. Three weeks after viral inoculation, the fifth leaf was photographed (A), and the viral RNA was quantified (B).

cDNA was prepared from RNA extracted from the fifth leaf of 5 individual plants, in two separate experiments (N=10). CT value for each virus is indicated, as compared with CT values for the housekeeping gene TIP41. Bars represent mean ±SE, all points shown.

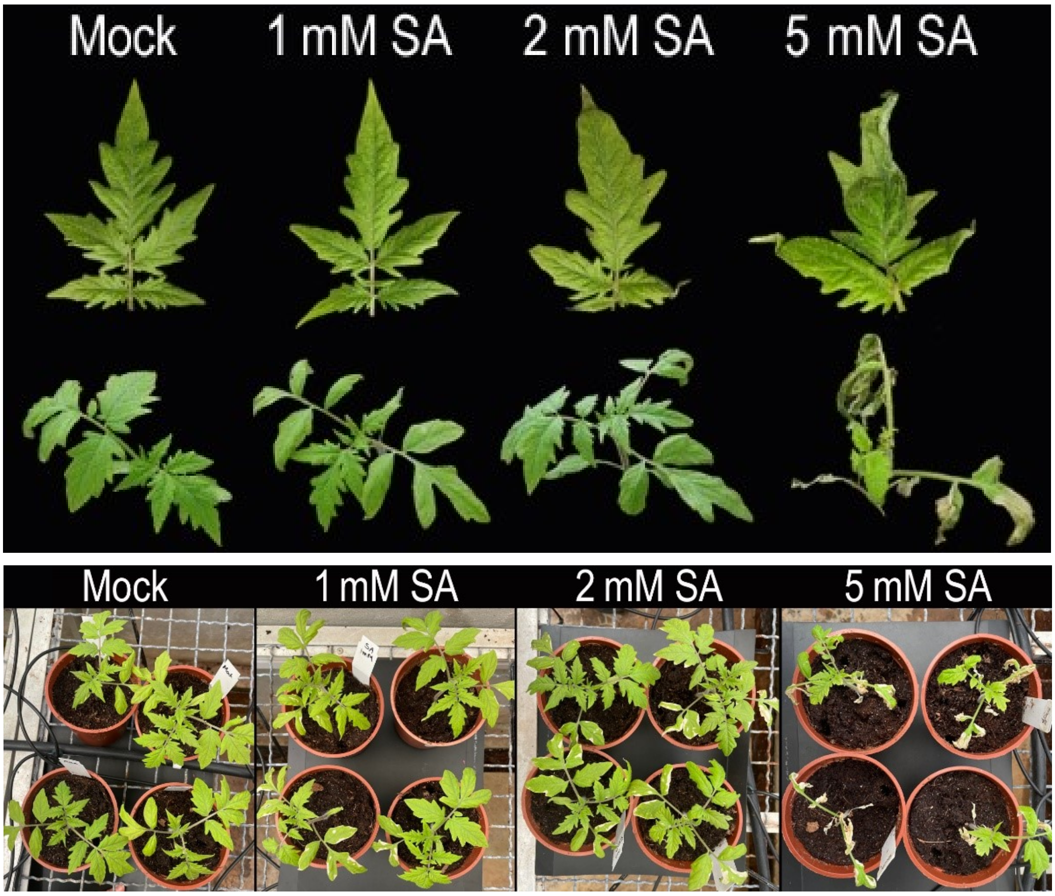

<u>Figure S2</u>: 5mM SA is phytotoxic.

2-week-old *S. lycopersicum* cv. Moneymaker plants were sprayed with indicated concentrations of SA for two weeks (4 treatments). Plants and third leaves were photographed after the fourth treatment, except in the case of 5 mM, which was photographed after the second treatment.

Table S1: Primer pairs used in qRT-PCR

Gene	**Accession No.**	**Primer pairs (5'-3')**
PR1a ***(Pathogenesis related-1a)***	Solyc01g106620	F: CTGGTGCTGTGAAGATGTGG<br>R: TGACCCTAGCACAACCAAGA
PR1b ***(Pathogenesis related-1b)***	Solyc00g174340	F: GTGTCCGAGAGGCCAAGCTA<br>R: AGGACGTTGTCCGATCCAGTT
Pti-5 ***(Pto-interacting 5)***	Solyc02g077370	F: GACATGGTGCGAGAGTATGG<br>R: CTGAAACAGAGGCGTTCACT
ERF-1 ***(Ethylene-responsive factor 1)***	Solyc05g051200	F: ATTAGGGATTCAACGCGTAA<br>R: AGAGACCAAGGACCCCTCAT
LoxD ***(LipoxygenaseD)***	Solyc03g122340	F: CCATCCTCACCACCCTCATC<br>R: TACTCGGGATCGTTCTCGTC
NPR-1 ***(Non-expressor of PR proteins)***	Solyc07g040690	F: ACAAGTTGATGGCACGTCTG<br>R: CCGATTCAAGTGCTCCTCTT
JAZ ***(Jasmonate-Zim Domain)***	Solyc12g009220	F: AAAGGTTCTGTTGGAGATCTTAATAATC<br>R: ACGATGACGACGATGAATCAC
PAL ***(Phenylalanine ammonia lyase)***	Solyc09g007900	F: TCAAGGCAGCTCAGAAGCTC<br>R: CTTAGTTGCTGCACGGATGA
CYP ***(Cyclophillin)***	Solyc01g111170	F: TGAGTGGCTCAACGGAAAGC<br>R: CCAACAGCCTCTGCCTTCTTA
RPL8 ***(Ribosomal protein L2)***	Solyc10g006580	F: TGGAGGGCGTACTGAGAAAC<br>R: TCATAGCAACACCACGAACC
Tip41	Solyc10g049850	F: ATGGAGTTTTTGAGTCTTCTGC<br>R: GCTGCGTTTCTGGCTTAGG
ToMV	KR537870.1	F: CTACGGTTGCAATTCGGTCT<br>R: AGGTCCAGACCAACCCAGACAT
ToBRFV	KX619418.1	F: TGGCCGGTCTGTCTGGAGAC<br>R: GGGGCCCGTGTACACAATCG
